# Reflections on “Building Back Better” Child and Adolescent Mental Health Care in a Low-Resource Postemergency Setting: The Case of Sierra Leone

**DOI:** 10.3389/fpsyt.2019.00758

**Published:** 2019-10-31

**Authors:** Hélène N.C. Yoder - van den Brink

**Affiliations:** Department of Sociology and Anthropology, Institute for Social Science Research (AISSR), University of Amsterdam, Amsterdam, Netherlands

**Keywords:** Sierra Leone, children, adolescents, public mental health, postemergency, Interactive Systems Framework

## Abstract

Over the past three decades, Sierra Leone has experienced two major humanitarian crises: an armed conflict (1991–2002) and an Ebola virus disease outbreak (2014–2015). In addition to these country-wide crises, the capital Freetown experienced a mudslide affecting thousands of people in 2017. In response to these emergencies, donors and aid organizations showed an increased interest in supporting and implementing mental health and psychosocial support interventions. Despite these efforts, the mental health infrastructure of the country remains frail. Specifically, systemic improvements in the implementation of evidence-based mental health care for children and adolescents appear to be lacking. In this article, the Interactive Systems Framework for Dissemination and Implementation is used as a tool to analyze issues related to the development of a sustainable, contextually relevant child and adolescent mental health-care delivery system. The author draws on her long-term experience as a child mental health specialist in Sierra Leone. Observations and hypotheses are tested and supplemented by formal and informal reports and national and international literature. The three systems described by the Interactive Systems Framework are explored in the context of Sierra Leone: (1) Synthesis and Translation, (2) Support, and (3) Delivery. Interaction between the three Systems is discussed as critical to the successful dissemination and implementation of interventions. Ample attention is given to contextual factors that are believed to be paramount to the development of child and adolescent mental health care in Sierra Leone. The article concludes with a reflection on the usefulness of the Interactive Systems Framework in the dissemination and implementation of child and adolescent mental health-care interventions in low-resource, postemergency settings. It is suggested that, in addition to funding and policies, the child and adolescent mental health system in Sierra Leone could benefit from the development of contextually relevant interventions, improvement of capacity-building efforts, and acknowledgment of the role of community-based practitioners in the delivery of services. Local mental health experts, especially those trained in child and adolescent mental health, should be empowered to work together with culturally competent expatriate professionals to improve child and adolescent mental health care in Sierra Leone.

## Introduction

Around the world, lower- and middle-income countries are disproportionately affected by natural and manmade disasters (1, 2), ^1^ creating increased mental health needs in countries that often already have under-resourced mental health systems. In 2013, the World Health Organization (WHO) published the report “Building Back Better: Sustainable Mental Health Care after Emergencies” (3). The document argues that, while disasters create much adversity, humanitarian settings also present opportunities that are often lacking in regular development settings. The increased attention for mental health after an emergency often raises the interest of policy makers and increases the political will to make significant changes to the mental health system. Both national and international agencies frequently respond with increased resources to develop mental health and psychosocial support (MHPSS) services for the affected population. The “Building Back Better” report presents 10 lessons learned and key overlapping practices from areas that seized the opportunity to build quality and sustainable mental health systems in the aftermath of emergencies (see **Box 1**).

Box 1 Building Back Better: Lessons Learnt and Key Overlapping Practices from 10 Low-Resource Settings.*

1. Mental health reform was supported through planning for long-term sustainability from the outset.
2. The broad mental health needs of the emergency-affected population were addressed.
3. The government’s central role was respected.
4. National professionals played a key role.
5. Coordination across agencies was crucial.
6. Mental health reform involved review and revision of national policies and plans.
7. The mental health system was considered and strengthened as a whole.
8. Health workers were reorganized and trained.
9. Demonstration projects offered proof of concept and attracted further support and funds for mental health reform.
10. Advocacy helped maintain momentum for change.

*) World Health Organization. *Building back better: sustainable mental health care after emergencies*. Geneva: WHO (2003)

Over the past three decades, the West-African country Sierra Leone has experienced two major humanitarian crises: an armed conflict (1991–2002) and an Ebola virus disease (EVD) outbreak (2014–2015). In addition to these country-wide crises, the capital Freetown experienced a severe mudslide in 2017. In response to these emergencies, multiple donors and international aid organizations showed an increased interest in supporting and implementing MHPSS interventions. However, despite efforts to put the “Building Back Better” recommendations into practice (4), the mental health infrastructure of the country remains under-resourced to this date. Specifically, systemic improvements in the implementation of evidence-based mental health care for children and adolescents appear to be lacking.

Children and adolescents make up a significant proportion of the population of Sierra Leone. Of the total population of seven million, ∼42% is under the age of 14 years and ∼60% is under the age of 25 years (5).^2^ There are no epidemiological data on child and adolescent mental health (CAMH) in Sierra Leone. However, using prevalence rates from similar contexts, the treatment gap for this population is estimated to be 99.8–99.9% (6). Current specialized mental health services for children and adolescents are limited to one outpatient clinic at the Ola During Children’s Hospital in the capital Freetown, where services are provided by a mental health nurse who was trained in CAMH in Nigeria (7, 8). Three other nurses and four medical doctors were trained through the same program but are yet to be deployed in this capacity by the Government of Sierra Leone (S.K. Conteh, personal communication, April 14, 2019). The 19 mental health nurses posted at the mental health units in district hospitals across the country received training in the Mental Health Gap Action Program (mhGAP) intervention guidelines (9), as did various other health professionals (7), but general supervision is minimal or absent and specialized supervision for services provided to children and adolescents unavailable. There are three psychiatrists (one expat) and two clinical psychologists who are mostly serving the adult population (M. van Leeuwen, personal communication, March 7, 2019).

In Sierra Leone, the Ministry of Health and Sanitation plays a central role in the governance of mental health-care delivery. To successfully implement comprehensive MHPSS services, they collaborate with other government ministries, most notably the Ministry of Social Welfare, Gender and Children’s Affairs. Government efforts are supplemented by nongovernmental organizations (NGOs), which mainly provide psychosocial counselling, often for specific groups such as girls affected by gender-based violence or vulnerable youth.

There have been previous evaluations in different contexts of how building back better may have succeeded or not (10, 11). In this paper, I use the Interactive Systems Framework to conduct a systematic analysis of the apparent lack of success in building back better from a dissemination and implementation perspective (12) and make recommendations to strengthen the development of a relevant CAMH system.

## Approach

Dissemination and implementation science is a relatively new field of study in global mental health which addresses the gap that often exists between evidence and practice. Dissemination refers to the methods or strategies that are used to transmit information on evidence-based interventions to end users, while implementation refers to the process of putting the evidence-based interventions into effect (12). The specific dissemination and implementation model applied here was selected from a review of 61 models for dissemination and implementation research by Tabak et al. (13). The Interactive Systems Framework (ISF) seemed to be most suitable for the purpose of the current analysis, as it is characterized by: (a) a broad construct flexibility (meaning it can be applied to a wide variety of dissemination and implementation contexts and activities), (b) an equal focus on dissemination and implementation, and (c) operation at multiple socioecological levels.

The Interactive Systems Framework was first described in 2008 by Wandersman and colleagues as “*a heuristic framework for organizing the theory, research, and practice (activities) of the dissemination/implementation process*” (14, p. 178). The ISF concentrates on three ‘Systems’ that work together to disseminate and implement interventions: (a) the Synthesis and Translation System, (b) the Support System and (c) the Delivery System. The three Systems are characterized by activities rather than specific organizations or individuals.

**Figure 1:**
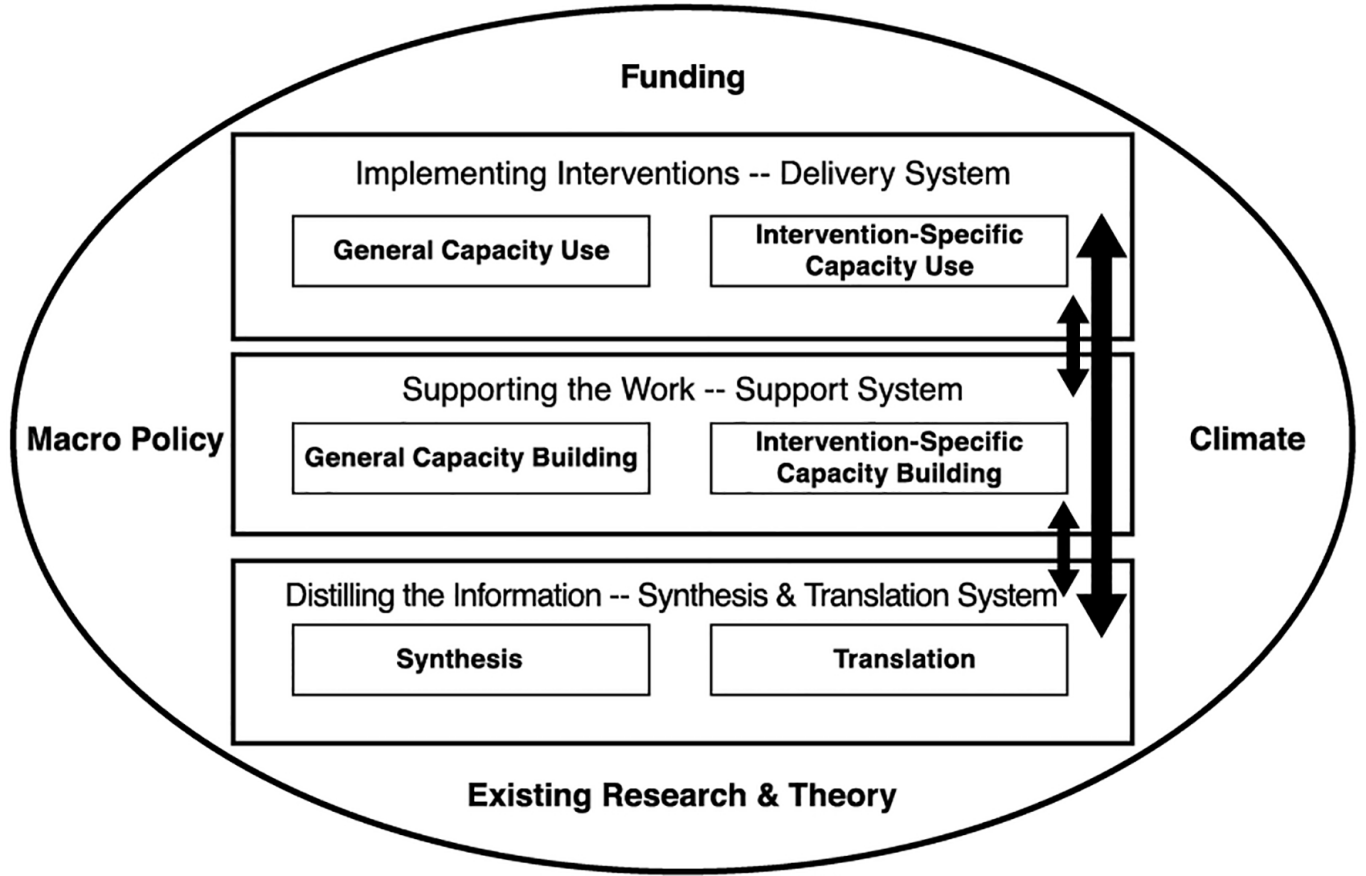
The Interactive Systems Framework (14).

The focus of activities within the “Synthesis and Translation System” is on translating scientific information generated through research into understandable and actionable information to be used by practitioners. The ISF does not prescribe the method used for this, but a collaborative effort of both researchers and end users is recommended (14). The “*Support System” carries out two support functions: (a) general capacity building (“intended to enhance the infrastructure, skills and motivation*” (14, p.175)) and (b) intervention-specific support (related to the implementation of a specific intervention). The “Delivery System” refers to activities focused on implementation. The individuals or organizations implementing mental health activities may have varying degrees of (a) general capacities (the skills to maintain a functioning organization and the capacity to connect with other organizations and the community) and (b) intervention-specific capacities (activities like identifying, implementing, and continuing interventions) (14). For the successful dissemination and implementation of interventions, it is essential that the three Systems work well together, communicating information and knowledge back and forth. The three Systems of the ISF are embedded in a wider context which is considered important but is not the main focus of the original framework (14). The developers identified the context elements of Existing Research and Theory, Climate, Funding, and Macro Policy. For the purpose of this paper, we describe “climate” as the historical, socio-political, mental health and cultural climate. **Figure 1** shows a visual representation of the ISF.

According to the developers, the ISF is primarily descriptive but is also meant to improve the dissemination and implementation process (14). While initially developed for the field of primary prevention, the ISF has also been applied in the fields of secondary and tertiary prevention in health and mental health (15–18). In 2018, it was for the first time successfully used in a low-resource context (19). As far as I am aware, it has never before been used to analyze a countrywide mental health system with the aim to improve the dissemination and implementation of CAMH interventions for an entire population.

For the analysis in this paper, I draw on my experience as an expatriate child mental health specialist in Sierra Leone (approximately 13 years over a period of 16 years). Observations and hypotheses following from this experience are tested and supplemented by both formal and informal reports and national and international literature on mental health-care development and delivery.

In the subsequent paragraphs I will first describe and analyze contextual factors, followed by an exploration of the three “Systems” within the ISF in Sierra Leone. The description of each System will include an analysis of the current situation and recommendations for System strengthening. An important aspect of the ISF concerns interaction between the three different Systems in the model. This will be addressed in the last paragraph, before a description of strengths and limitations of my approach and conclusions and recommendations.

## Context

Although contextual factors are literally placed on the periphery of the ISF (20), I believe they are paramount in understanding the development of CAMH care in Sierra Leone (cf. 21) and will therefore give more weight to contextual factors in my application of the ISF model.

### Climate

#### History and Sociopolitical Factors

The long-term history of Sierra Leone is deeply influenced by the slave trade. Many slaves in Europe and the USA originated from Sierra Leone. In 1787, after the abolition of slavery in the UK, a British naval base was established in Freetown in an effort to combat the slave trade and to provide a settlement for freed slaves (22).^3^ In 1961, the country gained independence from the UK, which had colonized the country since the early nineteenth century. Sierra Leone’s current population consists of 2 larger and 14 smaller ethnic groups. The two main religions are Islam (71%) and Christianity (27%) (23).^4^ From 1991 to 2002, the country was affected by a brutal armed conflict, caused by a complexity of factors including inadequate governance (24) and, as some argue, the marginalization of youth (25). The country’s rich diamond mines were sought after by many parties, persistently fueling the conflict (24). Approximately 50,000 people were killed, and more than half of the population was displaced (26). Many people were affected by human rights atrocities such as sexual violence and the amputations of limbs (27). It is estimated that nearly 7,000 children were recruited as child soldiers (28). The conflict affected and continues to affect the mental health of people across generations (29–31). In the 2014–2015 EVD outbreak, over 8,700 people were diagnosed with the disease, and nearly 4,000 died (32)^5^. The outbreak had a deep impact on the children of Sierra Leone. Participatory research with children across nine districts identified the closure of schools as their primary concern. Other concerns included the varied effects Ebola had on their lives (such as fear, anxiety, and grief), the reduced access to health care for other health problems, and the broader economic impact of the outbreak. The school closure was also believed to have led to an increase in child labor, exposure to violence, and teenage pregnancy (33). As the country’s health infrastructure was disrupted, many children failed to get their vaccinations (34), potentially increasing the spread of communicable diseases. In August 2017, a large landslide near the capital Freetown slipped into a river valley, exacerbating existing flooding in Freetown and surroundings. Approximately 6,000 people were affected, of which 1,141 were declared dead or missing. The disaster caused major damage and loss, affecting housing, infrastructure, health, sanitation, and education (35). Although no epidemiological data are available, it is safe to assume that the multiple crises and daily stressors related to poverty [over 60% of the population live below the national poverty line (36)^6^] have affected the mental health of the population in varying degrees (37, 38). Additionally, malaria, which is highly prevalent in Sierra Leone, increases the risk of developmental, cognitive, or behavioral disorders in children (39–41). Substance abuse and related mental disorders are frequently mentioned as a significant and possibly growing risk for adolescents in Sierra Leone (6, 42). Mental health issues related to gender-based violence are a concern for girl children. Accurate data are lacking, but in 2019, the President of Sierra Leone declared rape and sexual violence a national emergency (43).^7^

#### Mental Health Developments

At the end of the armed conflict, the country’s mental health system consisted of one psychiatric hospital, which dated back to the colonial days, and one psychiatrist. The years that followed were marked by strained relationships between national and expatriate actors in mental health. The only psychiatrist, who was also the Director of Mental Health Services at the Ministry of Health and Sanitation, for a while refused to work with the local and regional offices of the WHO and deeply distrusted the agenda of international NGOs (44), although this last issue was probably not unique for the mental health sector.

*Example: In the years after the war, it was common to hear people comment: “Those NGO people, they only drive around in their big vehicles, but we don’t see any results of what they are doing.”*

A strong emphasis on the uniqueness of “African psychiatry” was used to keep expat mental health professionals at a distance. In turn, expat professionals seemed to have a lack of cultural understanding which affected their interactions with the government (44). Over the years, new actors were added to the scene, relationships seemed to improve, interest in mental health increased, and important strides towards development were made. However, in an evaluation of the EVD outbreak MHPSS response in 2015, a significant critique still concerned the dominance of international organizations in mental health intervention decision making, leading to culturally insensitive work and a disenfranchisement of national actors (4). As Bah and colleagues observe, many postemergency MHPSS interventions *“remain relatively top–down and external. They focus on a very specific population and are often not well integrated into the health system at national, district and primary levels. This means that even those which have been well-evaluated have had a limited impact on the overall mental health and psychosocial wellbeing of the population of Sierra Leone”* (7, p. 44).

#### Cultural Factors

In the context of this paper, I will not attempt to give a comprehensive overview of the cultural values, practices, and beliefs that affect CAMH care development in Sierra Leone, especially when considering the diversity of cultures within the country. Nevertheless, four important aspects that should be highlighted are the following: (a) the importance of religion in mental health perceptions and help-seeking behavior (6), (b) the notion that mental health is commonly perceived as a collective issue rather than a personal one (44), (c) the stigma attached to mental health problems in Sierra Leone, which affects both people with mental disorders and those living or working with them (6, 45), and (d) the low status that children hold in Sierra Leone society (46), which may affect the priority given to child mental health.

### Macro Policy

Mental health care in Sierra Leone is guided by legally binding acts and government policies and strategic plans. The “Lunacy Act” (47), which dates back to the colonial days, is outdated and may contribute to alienation and discrimination of people with mental health problems (45). Its revision has been planned for many years but has not yet materialized. The first Mental Health Policy was launched in 2012 (48) but, according to Bah and colleagues, had minimal practical impact (7). A new Mental Health Policy was launched in 2019 (49). Although the Policy is an important condition for mental health development, the single reference to children as a “*special population*” (49, p. 6) and the absence of any reference to adolescents do not do justice to a group which makes up at least 50% of the population. After the EVD outbreak, the Ministry of Social Welfare, Gender and Children’s Affairs developed the MHPSS services packages, which describe the services that should be offered to all persons affected by crises, with a particular focus on children (50). The 2015–2018 MHPSS Strategy of the Ministry of Social Welfare, Gender and Children’s Affairs is currently under review. The Persons with Disability Act describes the rights and privileges of persons with disability, including those with mental impairments (51). According to this act, children should be screened for early signs of disabilities at health centers, and children with disabilities should have access to education and free health care. While some progress has been made towards these goals, in many areas the implementation is found deficient and hindered by a lack of referral systems (6).

### Funding

One of the most limiting factors in mental health-care development in Sierra Leone is funding (45, 52). Despite the increased interest in MHPSS after the EVD outbreak, there is no separate budget line for mental health in the budget of the Ministry of Health and Sanitation (7). Similarly, the Ministry of Social Welfare, Gender and Children’s Affairs depends exclusively on outside donors for the implementation of their MHPSS activities (J.A. Duncan, personal communication, July 30, 2019). Although international NGOs are often willing to invest in short-term psychosocial intervention projects, very few invest in the treatment and care of those who need specialized and prolonged mental health care (44). After the EVD outbreak, it was observed that the emphasis had been on care for Ebola survivors at the cost of resources for general population-wide mental health services (53). To date, the majority of health personnel trained in mental health (including CAMH) is not receiving increased monetary benefits, causing many to divert either part or full time to other areas of health care (M. van Leeuwen, personal communication, March 7, 2019).

### Existing Research and Theory

The existing research and theory that inform CAMH in Sierra Leone include international guidelines and studies on postdisaster MHPSS interventions and mental health research carried out in Sierra Leone. In the Introduction, I already mentioned the WHO “Building Back Better” document (3). Many of the post-Ebola interventions were guided by the IASC Guidelines on Mental Health and Psychosocial Support in Emergency Settings (54), the Mental Health and Psychosocial Support in Ebola Virus Disease Outbreaks: A Guide for Public Health Programme Planners (55), and the “Psychological First Aid during Ebola Virus Disease Outbreaks” manual (56). To guide general CAMH across the globe, the WHO published their “Caring for Children and Adolescents with Mental Disorders: Setting WHO Directions” (57). There is also a growing body of research on the effectiveness of CAMH interventions in low-resource humanitarian settings (58–61) and the dissemination and implementation of evidence-based mental health interventions in low-income countries (12, 62–64). With a few exceptions, the majority of CAMH research in Sierra Leone concentrates on children affected by the armed conflict or associated with the armed forces (6). However, valuable lessons can be learned from these and other studies in relation to cultural adaptation and the implementation of CAMH services for a variety of needs. This will be discussed in the next paragraph.

## Distilling the Information—Synthesis And translation System

In the field of global mental health, and MHPSS in humanitarian settings specifically, authors have noted major gaps in the synthesis and translation process (65–68). One assumed important aspect of the synthesis process is to identify core elements that appear to be responsible for an intervention’s effectiveness and which after adaptation will have to be implemented with fidelity to the original design (14). This is particularly challenging when translating knowledge originating primarily from Western, high-income countries to interventions that are feasible and relevant for children and adolescents in settings with low resources and different belief systems (69).

### Locally Relevant Synthesis and Translation Activities

Examples of synthesis and translation activities that have been particularly relevant for CAMH in Sierra Leone are the development of a Psychological First Aid (PFA) manual for EVD outbreaks by the WHO and several international NGOs (56) and a training manual for delivering PFA to Ebola-effected communities in Sierra Leone (70). While the PFA manual of the WHO gives ample attention to children and adolescents, the training manual on PFA for Sierra Leone has a strong bias towards adults, thus neglecting a significant part of the population. The manual has a section on “Tips/skills for good communication with children,” but the suggestions listed there are not specific for children and adolescents and important issues such as communication through play or art are missing. The list of stress symptoms provided does not include symptoms of stress common in children or adolescents, such as regressive behavior and changes in play in younger children, or acting out among adolescents. A training manual on basic psychosocial support in education published by the Ministry of Education, Science and Technology of Sierra Leone (71), however, contains an abundance of practical information on psychosocial strategies for children and adolescents in the classroom setting. Both locally adapted manuals do address the issue of shame, a complicated emotion which the author has found to be very prevalent in Sierra Leone and often overlooked and poorly understood by outsiders. Shame was also identified by one organization as a frequently cited MHPSS problem during the EVD outbreak (72). Lastly, a “Toolkit for Community PSS Workers” with accompanying Information Guide was developed by the international NGO Trocaire with members of the Psychosocial Support Working Group in Sierra Leone (50, 73). The strength of the Toolkit is that it was developed with local partners, but its usefulness is limited by the overemphasis on relaxation exercises (11 out of 23 tools) and the possible lack of fidelity to original interventions. For example, a narrative therapy intervention aiming at resilience building, which originally was designed to last at least 8 h, was reduced to a 45- to 60-min intervention with significant changes to important components (74). With the increased interest in a resilience approach in MHPSS interventions, the Resilience Measure for Children and Youth is an interesting tool which deserves to be further explored for use in Sierra Leone. Currently, it is not clear to what extent the tool has been adapted and tested in Sierra Leone, an extremely important process considering the complexity of the concept of resilience (75). It should be noted here that not all MHPSS strategies and interventions in the discussed documents have been tested for cultural relevance and effectiveness in Sierra Leone, which limits their value in terms of evidence-based practice.

### Cultural and Power Differences

Including end users in the translation process, as recommended by the ISF, needs to be done with sensitivity to the culture and with cognizance of the power differences between international organizations and local experts and the local community.

*Example: An international aid worker in the EVD outbreak response, with no previous experience in Sierra Leone and representing an organization with a significant budget, proposed a guided imagery exercise for children in which they imagine to be an animal. When she was told that this concept may not be appropriate in a culture where it is not uncommon for vulnerable children to be accused of changing into animals for witchcraft purposes, she was surprised that she had received no such feedback from the local authorities who gave permission for her work.*

It is possible that, similar to other contexts (76, 77), power differences in Sierra Leone are not just monetary or knowledge related but that there is also a deep-seated belief in many Sierra Leoneans themselves that their views are inferior to those of international workers, especially white expatriates. It is plausible that this belief dates back to the days of slavery and colonialism and has been reinforced by dependence on international aid over the last few decades.

*Example: Participants in a workshop were given pictures of people of different age, race, and socioeconomic status and were asked to rank them according to their “value.” The young white female was outranked only by the president.*

Empowerment of local experts is therefore an important process which requires time and sensitivity and which should be an integral aspect of mental health system development.

### Strengthening the CAMH Synthesis and Translation System

There are several things that can be done to strengthen the synthesis and translation system for CAMH in Sierra Leone: (a) Existing literature should be reviewed for culturally relevant tools, cultural themes, and recommendations that can inform and strengthen new CAMH interventions. **Table 1** gives a few examples identified by the author in a rapid informal review of literature on CAMH-related issues in Sierra Leone (6, 78–85). (b) Research from related fields should be reviewed for cross-cutting issues. For example, the Columbia Group for Children in Adversity conducted an ethnographic study in Sierra Leone on community-based child protection mechanisms, which includes information relevant to the development of MHPSS interventions for children and adolescents. Examples are views of childhood (e.g., children are defined in relational terms; young people who become sexually active are considered adults), power asymmetry in the implementation of interventions, and practical lessons learnt in relation to capacity building and logistics (86). (c) Input from end users should be improved with sensitivity to power relations and culture. Local professionals, especially those trained in CAMH, should be empowered in the field of synthesis and translation. (d) Together with end users, topics should be identified for further research and fed back to the research community. An example of this could be the exploration of shame as an important emotion in the context of mental health.

**Table 1:** Examples of relevant themes, tools, and recommendations.

Category	Description	Study
Theme	The importance of religion in mental health.	Stark (78) Behrendt (79) Yoder et al. (6)
Theme	Minimal evidence of reflecting on past. A desire to move on after trauma. Difficulty in verbal processing.	Burman & McKay (80)
Theme	Avoidance of emotional vulnerability.	Harris (81)
Recommendation	MHPSS interventions should address the specific needs of girl children.	Behrendt (79)
Recommendation	Apart from PTSD, also treat affect recognition, self-regulation and social engagement.	Ardizzi et al. (82) See also Betancourt (83)
Recommendation	Findings suggest that the inclusion of family-based elements will increase the impact of MHPSS interventions with adolescents.	Betancourt et al. (84)
Tools	The following tools were translated and tested for use with adolescents in Sierra Leone: Oxford Measure of Psychosocial Adjustment, World Health Organization Quality of Life-BREF instrument, Difficulties in Emotion Regulation Scale.	Newnham et al. (85)

## Supporting the Work—Support System

The support system carries out both innovation-specific and general capacity building.

### Innovation-Specific Capacity Building

Examples of innovation-specific support in Sierra Leone are the training of the government and NGO workers in postemergency interventions such as PFA, the training of personnel on various levels of the health system in the mhGAP Intervention Guide (9, 87),^8^ and short-term mental health training by international organizations or institutions with MHPSS projects in Sierra Leone. A popular international training concept that is also often used in Sierra Leone is the “training of trainers” approach, where participants in a training event are expected to replicate the training in their own environment (88). The author is not aware of any research done on the training of trainers concept in Sierra Leone in terms of replication, fidelity to the original design, and efficacy or effectiveness. However, the local practice of paying people for attending training (usually in the form of a generous travel stipend and provision of food and lodging) may limit the probability that training will be replicated, as few local organizations will have the funds to pay participants.

Ray et al., who studied the support system of the ISF in the context of evidence-based prevention projects, describe how literature increasingly shows that training by itself is not sufficient to ensure quality implementation and that it should be accompanied by ongoing tailored support of those who participated in the training (88).While many organizations provide short-term MHPSS training in Sierra Leone, there is frequently no sufficient follow-up in the form of ongoing coaching or supervision (52), nor are there clear guidelines for supervision in terms of qualifications of the supervisor, frequency and method of supervision, etc. (89). This is often because of a lack of funding or human resources. This is especially a concern in clinical mental health, where practicing skills under the supervision of a senior professional is a crucial aspect of training (90, 91). An example of how this could be done is the apprenticeship model for the training of local providers of mental health interventions as described by Murray et al. (92).

### General Capacity Building

In the aftermath of emergencies in Sierra Leone, the emphasis has often been on innovation-specific capacity building. However, as Noonan and colleagues point out, while innovation-specific capacity building is important, by itself it does not predict sustainability. The general capacity of the organization or setting in which the intervention is embedded may be even more important for its continuation (93). General capacity building, which is described by Wandersman and colleagues as intended to improve “*infrastructure, skills and motivation*” (14, p. 175), has often been overlooked.

A successful project aimed at general capacity building has been the Mental Health Leadership and Action Program, which was launched in 2010 in the five Anglophone countries in West Africa, including Sierra Leone. The program trains people from various professional backgrounds in mental health leadership and advocacy (building skills and motivation) and supports the establishment of stakeholders councils (building infrastructure) (94). In Sierra Leone, the program supports the Mental Health Coalition, an advocacy organization bringing together stakeholders including service users, practitioners, national and international NGOs, civil society, traditional healers, researchers, and human rights organizations (45, 95).^9^ Despite the general success of the Mental Health Leadership and Action Program and the Mental Health Coalition in advocacy for mental health, neither program has been able to successfully address the lack of government attention to CAMH.

The Youth Forward intervention, developed by Betancourt and colleagues following 15 years of longitudinal research on the mental health of young people affected by the armed conflict in Sierra Leone, is a promising example of a CAMH intervention, which takes both innovation-specific and general capacity building into account (83). The program is an attempt to scale up the Youth Readiness Intervention, which was developed for war-affected youth, and will be implemented as an integrated part of a youth employment program. It recognizes the need to move away from remote expertise and to cultivate local knowledge using a collaborative approach to develop a core of local experts across agencies. Apart from innovation-specific knowledge, the core team develops “*critical skills related to collaboration, leadership, communication and quality improvement*” (83, p. 32). The effectiveness of this approach has not yet been evaluated. If successful, it could be an interesting model to be replicated, possibly in other sectors, as it remains to be seen how sustainable mental health initiatives in the employment sector will be.

### Educational Challenges

Any activity within the support system in Sierra Leone is affected by the country’s educational system which, from primary to tertiary level, has been severely affected by the armed conflict and again by the EVD outbreak. Large numbers of teachers do not have educational qualifications (96). At both primary and secondary levels, learning outcomes are often threatened by poor basic literacy and numeracy skills and factors such as absenteeism and large class sizes (97). The author has noticed that many students in tertiary education have limited access to academic resources. While many people are eager to learn, the general lack of quality education frequently affects their ability to benefit from certain forms of training.

*Example: During a workshop people were divided into smaller groups and given case studies to read and discuss. The case study described a familiar local situation. The author, who participated in the workshop, found herself in a small group with a school administrator and a teacher. Both struggled to read the full-page story and needed additional time to read it again for full comprehension.*

Shackman and Price, in their evaluation of a mental health project in rural Sierra Leone, describe how the course level of the project had to be adjusted for participants with lower literacy skills, affecting outcomes related to capacity (98). One of the requirements for the 18-month diploma course for mental health nurses at the College of Medicine and Allied Health Sciences in Freetown is to have at least five credits (50–64%) on the West African Senior School Certificate, a prerequisite that many struggle to fulfill. There are differing opinions on whether the college should lower its requirements or whether it is better to train less mental health nurses with better qualifications. (H. Lind, personal communication, April 4, 2019).

### Strengthening the CAMH Support System

Based on the above observations and earlier research (6), I recommend that the following measures are taken to strengthen the CAMH Support System in Sierra Leone: (a) The basics of CAMH should be integrated into the curricula of all health workers, thus increasing general awareness in the health system as a whole. (b) Those with interest in CAMH should have local access to specialized training. (c) Health professionals trained in CAMH should be empowered to educate other health professionals. (d) CAMH training should be practical and followed by systematic supervision. (e) Without compromising educational standards, training programs should cater to the educational levels of trainees, which may require an extended timeframe. (f) To promote inclusion of CAMH in policy and programming, leaders and policy makers in mental health should be made aware of CAMH needs and be trained in a life-course approach to mental health service development (99). (g) Organizations that are planning CAMH interventions in Sierra Leone should acquaint themselves at an early stage with general capacity needs of the setting in which the intervention will be implemented. If needed, provision should be made for general capacity building. (h) Training methods should be evaluated for their effectiveness in Sierra Leone.

## Implementing Interventions—Delivery System

The delivery of MHPSS services for children and adolescents may take place at different levels, from community-based services to specialized care at a national level, as described in the WHO Pyramid Framework (100). Similar to the support system, the delivery system is characterized by innovation-specific and general capacities.

### Intervention-Specific Capacity

Intervention-specific capacity for children and adolescents in Sierra Leone is mostly limited to psychosocial support on the community level, which is usually provided by national or international NGOs, and often disproportionately directed towards the Western (urban) areas of Sierra Leone, as was noted in the EVD MHPSS response (4). While important, the author has observed that many of these interventions are dependent on donor funding and not integrated in the health, educational, or religious systems of the country—as seen in other humanitarian settings (67). This limits their sustainability. On the district level, government mental health nurses and a limited number of community health officers and medical doctors trained in the mhGAP Intervention Guide, in an effort to task share (101), should be able to provide care for children and adolescents with emotional, behavioral, or developmental disorders. However, the quality of care these health workers can provide may not be adequate due to the limited training they have received in CAMH and the absence of specialized supervision. Most childhood disorders, especially in younger children, are recommended to be managed without pharmacological treatment, especially in nonspecialist settings (9). Psychological interventions for children and adolescents require professional skills that likely take more time to learn and develop than most short courses can offer. They also require more patient contact time than most health-care workers at this level can offer, as the majority of them are expected to fulfill their other duties in health care as well. In the opinion of the author, this could be a limiting factor of the task-sharing model, which will affect its applicability in the CAMH sector. For people living outside the capital, travel distance and expenses make it nearly impossible to attend the country’s only CAMH clinic at the children’s hospital in Freetown.

### General Capacity

The general capacity of the delivery system refers to the capacity to maintain a functioning organization and to connect with other organizations and the community. The aforementioned educational situation in Sierra Leone may be a limiting factor for the general capacity in some organizations. In their evaluation of a mental health project, Shackman & Price (98) describe concerns related to program management, such as issues with day-to-day management, record keeping, report writing, liaising with other agencies, representation of the program at a senior level, monitoring and evaluation, etc.

Another major issue in the general capacity of the delivery system is the difficulty in retaining personnel. Local capacity gets depleted when personnel trained by NGOs in MHPSS do not find similar employment after the NGO’s activities are closed down (44, 98). We already mentioned that many mental health nurses are finding alternative employment, as the government has not yet accredited their training or increased their benefits and only one out of the eight people trained in CAMH is currently employed in a relevant position.

### Relationship With the Community

General capacity also refers to the capacity to relate to the community in which the intervention takes place. For interventions to be successful, it is important that the community owns and supports the intervention and makes appropriate referrals. Furthermore, there are actors in the community that may not be considered part of the professional workforce but that nevertheless play an important role in providing care. Kleinman describes these as the popular sector (individual, family, and community) and the folk sector (nonprofessional healing specialists) (102). In Sierra Leone, the last sector is represented by religious institutions such as churches and mosques and traditional healers. In her research on psychosocial needs of children without parental support in the eastern district of Kailahun after the armed conflict, Behrendt noted that many children found consolation in faith and faith-related activities (79). In line with local explanatory views which explain child mental disorders mostly in spiritual or supernatural terms, parents of children with mental health problems usually seek help from Christian healing ministries or traditional (often Muslim) healers. Owing to their large numbers, they are also more accessible than formal mental health-care services (6). It is the author’s impression that there is less stigma attached to seeking help from religious leaders than from mental health professionals. The relationship between professional mental health care providers and traditional or religious care providers in Sierra Leone, however, remains complex and in need of further exploration (98). While nonprofessional practitioners are accepted by the community and may have good intentions, their treatments are generally costly and frequently include harmful practices (6, 7).

*Example: A 5-year-old with symptoms of potential autism spectrum disorder presented to the hospital with burn scars on his abdomen, which were sustained during a healing ritual by a traditional healer.*

Example: Children who are felt to be witches (often based on their deviant behavior) are submitted to extended periods of “dry fasting” (fasting from food and liquids) before being “delivered from evil forces” at a church camp.

Despite reservations related to abusive practices, the important role of the faith-based healers cannot be denied, and for this role to be adequately studied and utilized, the actors deserve a recognized place in the delivery system. I will come back to this in the Discussion.

Support from the community and community-based practitioners is also important to address stigma.

*Example: A child with brain injury due to malaria regularly receives beatings from community members as she unintentionally picks up their belongings and misplaces them.*

Example: Neighbors tell a boy with cerebral palsy not to look at them for fear that eye contact with him may negatively affect their wellbeing. His family has to move frequently until the mother is able to build a small home on the outskirts of town.

Addressing stigma in the community is one aspect of an ecological approach which increasingly is considered to be preferential to an individual approach in MHPSS interventions for children and adolescents (54, 69, 103, 104).

### Measuring Effectiveness

To evaluate the effectiveness of CAMH interventions, there is a need for robust measurements that consider context and culture.

*Example: During the EVD outbreak, an international NGO performed a baseline assessment of the psychosocial wellbeing of a group of children before testing an intervention. The children were occupying desks in a classroom, with their parents sitting at the back of the room. When the NGO worker called out the various questions and asked the children to rate their experiences on a 1–10 scale, the parents called out to their children: “Write 7!,” “Write 5!” etc.*

Possibly influenced by earlier encounters with relief organizations in the context of emergencies, the parents seemed to expect that the benefits to be received from the NGO would be influenced by their child’s answers to the survey.

For CAMH interventions to be relevant to the culture, effectiveness should also be measured in a way that reflects cultural values. In their article on girls formerly associated with armed forces in Sierra Leone, Stark and colleagues (105) remind us that in many African contexts “*physical and mental health are often conceived of in relation to one’s environment, one’s ancestors and one’s relationship with others*…” (105, p. 4). Stark describes how participatory ranking was used to develop culturally relevant indicators for reintegration and well-being. Similar methods could be used to develop indicators for the effectiveness of new and existing CAMH interventions.

### Strengthening the CAMH Delivery System

The CAMH delivery system can be strengthened in various ways: (a) Organizations implementing MHPSS interventions for children and adolescents should be encouraged to integrate their activities into existing structures for sustainability. (b) Health workers trained in CAMH should be accredited and deployed across the country, thus decentralizing care. (c) Relationships between formal providers and the community should be strengthened to reduce stigma and abuse and promote adequate and mutual referrals. (d) CAMH interventions should be evaluated with robust measurements that are sensitive to the context and culture.

## Interactions

For innovations to be successfully disseminated and implemented, interaction between the three systems of the ISF is crucial.

*Example: The Toolkit for PSS workers was developed by multiple MHPSS partners soon after the EVD outbreak. About a year later, the landslide near Freetown took place, affecting thousands of people in the Western Area. A CAMH expert who participated in the MHPSS coordination meetings recalled that the Toolkit was never mentioned as a potential resource for interventions. She herself only found out about the Toolkit another year later.*

One of the distinctive features of the ISF is that it acknowledges the importance of a two-way flow of information rather than a top–down approach from research to practice (106). The interaction between the three systems is probably the least investigated aspect of the ISF, but could potentially be just as or even more important than the three individual systems (15).

In Sierra Leone, the different Systems in the dissemination and implementation process are separated by more than the typical differences in professional viewpoints and capacities. The Synthesis and Translation System and the Support System are dominated by international aid and relief organizations and experts, while the Delivery System consists mostly of local practitioners, who are often employed by the local government or local institutions. Interactions between the Systems are therefore also influenced by differences in cultural backgrounds, belief systems, goals, and expectations. For example, objective evaluation of trainings or interventions can be complicated by the fear of losing financial support or the cultural importance of giving socially acceptable answers or “saving face.” Van Gog, in her ethnographic study of the Sierra Leone Psychiatric Hospital, describes a typical meeting between international NGO workers and local government representatives. She observes: “*While in Sierra Leone culture, keeping up appearances is considered a virtue and problems are solved behind the scene, the Western NGO workers in the audience were eager to openly discuss the problems in the field of mental health care in order to work towards solutions there and then*.” (44, p. 68) The meeting ended in an impasse (44).

Puddy and Hall identify the arrows connecting the three systems in the ISF as “knowledge brokers,” which work between and across systems, have a crucial understanding of the context (sociopolitical/mental health/cultural climate, policies, funding, and research), and facilitate a flow of pertinent information between the systems. They serve the needs of the knowledge producers as well as the end users (107). An institutionalized brokerage approach was used in East Africa in an effort to close the gap between health research and policy (108). While a formal approach to knowledge brokering may be a step too far for CAMH in Sierra Leone, the author thinks that the Mental Health Coalition of Sierra Leone may be able to play an important role in facilitating knowledge brokering across the systems, as one of the Coalition’s goals already is to act as an advisory body on issues of mental health in Sierra Leone (45).

## Limitations and Strengths

A critical analysis of a country’s mental health system by one person has obvious limitations. The strength of being a long-term participant and observer in mental health can become a limitation when the author develops personal biases or blind spots that hinder an objective perspective. In addition, after the EVD outbreak, the author spent 2 years out of the country, after which she returned to work in a remote rural area in Sierra Leone. There, she experienced first hand the reality of being relatively isolated from many deliberations and activities related to national mental health care development, which is a potential source of bias. While these are important limitations, the author spent most of her years working with community-based organizations and speaks the lingua franca of the country, which has hopefully increased her contextual and cultural awareness. To minimize the limitations of a one-person approach, the author has had frequent discussions with local and international colleagues in the field of mental health, and extensively consulted with gray and international literature to verify and test her observations. To increase validity, the article was also reviewed by two Sierra Leonean mental health experts, including a physician trained in CAMH.

## Conclusions and Recommendations

In this paper, I have used the ISF to analyze CAMH service development in Sierra Leone. In this last section, I will evaluate the usefulness of the ISF in this type of exercise and highlight some of the issues that were identified to strengthen the dissemination and implementation of CAMH interventions in Sierra Leone.

**Figure 2:**
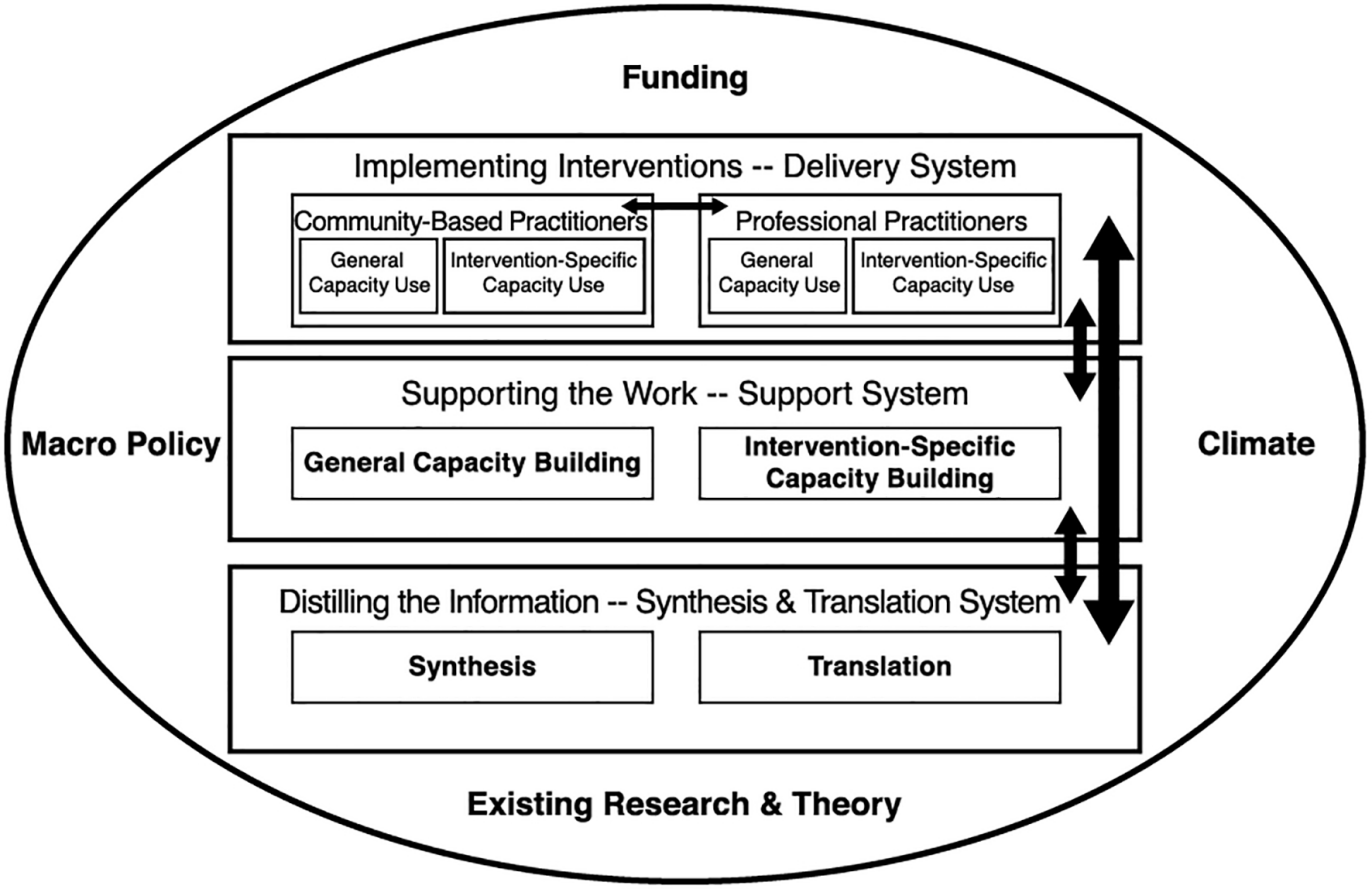
The Interactive Systems Framework (14) with Inclusion of Community-Based Practitioners.

Based on the analysis in this paper, I propose that the ISF can be a useful framework to analyze and guide the dissemination and implementation of CAMH interventions in lower- and middle-income countries. It shows the need for comprehensive policy making, planning, and funding, taking into account the various activities that bridge the gap between science and practice. The ISF could be even more useful if the role of community-based practitioners were to be acknowledged in the delivery system. In an altogether different context, Firesheets and colleagues’ main criticism of the ISF is that the role of the community in the delivery system “*is implied but not clearly defined*” (109, p. 354). In line with their suggestion, I propose to divide the Delivery System into Community-Based Practitioners (traditional and religious healers) and Professional Practitioners, each representing their own general and specific capacity (see **Figure 2**). An added benefit of the visualization of the Community-Based Practitioners in the framework is that it may encourage an increase in community-centered approaches (110) in addition to the more commonly used top–down or research-driven approach for the development of interventions. Effective elements in the services provided by Community-Based Practitioners could be identified and developed into contextually relevant interventions that supplement the efforts of Professional Practitioners. Both groups of practitioners could learn to appreciate each other’s contribution to the Delivery System and collaborate and refer for the benefit of children and adolescents with mental health problems. Whether the two groups need to be supported separately by parallel support systems remains something to be discussed.

In addition, considering the importance of the cultural context in which CAMH is developed, I suggest, similar to Gregory and colleagues (20), an increased emphasis on cultural competence in the ISF, both on the three System levels and in knowledge brokering. A modification of the model in this respect does not seem necessary as long as culture is included as an important aspect of the surrounding context.

While the ISF is a useful structure to optimize dissemination and implementation, as Lesesne and colleagues note, it does not offer solutions for the challenges that can arise from the context, such as funding or policies (15). Lack of funding and the absence of suitable policies continue to be major hindrances to the development of CAMH services in Sierra Leone. This leaves a challenge for the government and international donors. With the high percentage of children and adolescents in the population of Sierra Leone, it is surprising that so little attention is given to child and adolescent mental health. Quoting Bronfenbrenner, Betancourt and Kahn point out that the position and priority that children and those who work with them have in macrosystems determines how they are treated across ecological settings. (103) It seems that the lower status that children hold in Sierra Leone society has led to a neglect of their mental health needs on multiple levels. Advocacy by, with, and on behalf of children and adolescents is needed to give a voice to this significant part of the population. Although policy reform does not always translate into implementation (45), I agree with Belfer that the development of a specific CAMH policy is essential for the development of a nation’s CAMH system (111). In addition to funding and policies, CAMH care development in Sierra Leone could benefit from the development of contextually relevant CAMH interventions, improvement of innovation-specific and general capacity building, and acknowledgement of the role of Community-Based Practitioners in the delivery of CAMH services. Local mental health experts, especially those trained in CAMH, should be empowered to work together with culturally competent expatriate professionals on all three system levels of the ISF to improve CAMH care in Sierra Leone.

## Author Contributions

The author confirms being the sole contributor of this work and has approved it for publication.

## Funding

The Open Access publication fees for this article were funded by the Amsterdam Institute for Social Science Research, University of Amsterdam.

## Conflict of Interest

The author declares that the research was conducted in the absence of any commercial or financial relationships that could be construed as a potential conflict of interest.

## Footnotes

1
https://www.preventionweb.net/risk/poverty-inequality

2
https://www.cia.gov/library/publications/the-world-factbook/geos/sl.html

3
https://unipsil.unmissions.org/about-sierra-leone-history

4
http://worldpopulationreview.com/countries/sierra-leone-population/

5
https://www.cdc.gov/vhf/ebola/history/2014-2016-outbreak/index.html

6
http://www.sl.undp.org/content/sierraleone/en/home/countryinfo.html

7
https://statehouse.gov.sl/president-julius-maada-bio-declares-rape-and-sexual-violence-as-a-national-emergency-in-sierra-leone/

8
https://afro.who.int/news/improving-access-mental-health-services-sierra-leone

9
https://mentalhealthcoalitionsl.com/

## Abbreviations

CAMH, Child and adolescent mental health; EVD, Ebola virus disease; ISF, Interactive Systems Framework; MHPSS, Mental health and psychosocial support; NGO, Nongovernmental organization; PFA, Psychological first aid; WHO, World Health Organization.
